# Severe hyperkalemia following adrenalectomy for aldosteronoma: prediction, pathogenesis and approach to clinical management- a case series

**DOI:** 10.1186/s12902-016-0121-y

**Published:** 2016-07-27

**Authors:** A. Tahir, K. McLaughlin, G. Kline

**Affiliations:** 1 Department of Internal Medicine, Cummings School of Medicine- University of Calgary, Alberta, Canada; 2 Department of Nephrology, Cummings School of Medicine- University of Calgary, Alberta, Canada; 3 Department of Endocrinology, Cummings School of Medicine- University of Calgary, Alberta, Canada

**Keywords:** Case report, Adrenalectomy, Hyperkalemia, Aldosteronoma

## Abstract

**Background:**

As the field of Primary Aldosteronism (PA) becomes ever expanded, diagnosis of PA is increasingly diagnosed by endocrinologists. With increased PA screening, many of the cases are now found in patients with complex co-morbidities in addition to their hypertension. Post adrenalectomy renal impairment with hyperkalemia is now increasingly seen in these complex patients, as evidenced by the numerous reports on this issue that have appeared within the past 3 years. We present a small case series to illustrate the breadth of the problem, along with a discussion about how such CKD/hyperkalemic events may be predicted.

**Case presentation:**

We present three cases of primary aldosteronism with long standing hypertension (more than 10 years) hypokalemia (2.0–3.0 mmol/l). Serum aldosterone was high with low renin activity leading to high aldosterone to renin ratio (ARR). They underwent abdominal CT scan revealing adrenal mass and adrenal vein sample confirmed lateralization. None of the patients had evidence of renal disease before surgery (as evident by normal eGFR and serum creatinine). Post adrenalectomy they had reduction in the blood pressure and became eukalemic. Serum aldosterone and renin activity were low leading to a low ARR. Case 1 developed hyperkalemia and increased serum creatinine 6 weeks post operatively which resolved with initiation of fludrocortisone and every attempt to discontinue fludrocortisone resulted in hyperkalemia and rising creatinine. Her hyperkalemia is under control with oral sodium bicarbonate. Case 2 developed hyperkalemia and increasing creatinine 2 months post operatively transiently requiring fludrocortisone and later on managed with furosemide for hyperkalemia. Case 3 developed renal impairment and hyperkalemia 2 weeks post operatively requiring fludrocortisone.

**Conclusion:**

Post APA resection severe hyperkalemia may be a common entity and screening should be actively considered in high risk patients. Older age, longer duration of hypertension, impaired pre-op and post-op GFR and higher levels of pre-op aldosterone and are all risk factors which predict the likelihood of developing post-operative hyperkalemia. Fludrocortisone, sodium bicarbonate, loop diuretics and potassium binders can be used for treatment. Treatment choice should be tailored to patient characteristics including fluid status, blood pressure and serum creatinine. Potassium binders should be avoided in patients with history of recent abdominal surgery, opioid use and constipation. Serum electrolytes and creatinine should be monitored every 1–2 weeks after starting treatment to ensure an adequate response. Prolonged management may be necessary in some cases and at-risk patients should be counselled as to the meaning and importance of post-operative changes in measured renal function and potassium.

## Background

Primary aldosteronism (PA) is characterized by hypertension, suppressed plasma renin levels, inappropriately high aldosterone secretion and in some cases hypokalemia. PA accounts for about 10 % of hypertensive patients [1, 2] who are known to have a higher risk of cardiovascular disease as compared to patients with essential hypertension. For those with an Aldosterone producing adenoma (APA), adrenalectomy offers a high rate of possible cure.

Moderate-to-severe hyperkalemia post adrenalectomy has been described in the literature but the risk factors and outpatient management is not very well delineated. We describe three cases of severe post adrenalectomy hyperkalemia requiring complex and long term therapy and discuss the known risk factors for developing post APA-resection hyperkalemia and suggest an approach to outpatient endocrinology management.

### Case 1

A 51 year old woman with a 12 year history of hypertension developed hypokalemia (as low as 2.2 mmol/L). Her blood pressure was controlled with amlodipine 10 mg once daily and she received potassium supplements to maintain eukalemia. Her serum aldosterone was 2832 pmol/l with undetectable plasma renin activity. Her aldosterone to renin ratio (ARR) was therefore estimated at greater than 28,000 pmol/l/ng/ml/h (normal less than 2000 using renin activity of 0.1 ng/ml/h to avoid over-inflation). Her serum creatinine was 75 umol/l with eGFR of 85 ml/min. Abdominal CT showed a 3.5 cm low density left adrenal mass and adrenal vein sampling confirmed left lateralization with lateralization index values of 23:1 and 28:1 pre and post cosyntropin infusion. She underwent left adrenalectomy for what was reported as a 15.6 g adrenocortical adenoma on pathology. The post-operative biochemical course is presented in Table 1. At 2 weeks post-operative follow up, her blood pressure was 124/80, serum potassium 4.5 mmol/L and serum creatinine 52 umol/l without any medications. Her serum aldosterone was less than 70 pmol/l and renin activity of 0.36 ng/ml/h yielding ARR less than 194. However, at 6 weeks post-operative, she presented with serum potassium of 6.7 mmol/l, serum creatinine of 152 umol/l associated with persistent diarrhea (determined to be non-infectious) and clinical volume depletion. After fluid resuscitation she was started on fludrocortisone 0.1 mg daily and up-titrated to 0.1 mg bid over a 1-week period. At the higher fludrocortisone dose she became normokalemic and her serum creatinine decreased to 134 umol/l (Table 1). One month later, her serum potassium continued to be 4.8 mmol/l and serum creatinine 112 umol/l and so a dose reduction in fludrocortisone was attempted but at 0.1 mg per day, her potassium promptly rose to 5.6 mmol/l with creatinine 140 umol/l. Hyperkalemia persisted in the fluid replete state. She was not on any medications causing hyperkalemia. In the context for fludrocortisone responsive hyperkalemia we did not calculate TTKG (trans tubular potassium gradient). The fludrocortisone dose was increased again with similar normalization of potassium and drop in serum creatinine but her resultant blood pressures rose to 160/100.

**Table 1:** (Case1) Changes in Serum creatinine, potassium and blood pressure relative to medications. (Abbreviations: Bicarb- Bicarbonate)

Follow up period	Drugs	Creatinine umol/l	Potassium mmol/l	Blood pressure
2 weeks	None	52	4.5	124/80
6 weeks	None	152	6.7	-
10 weeks	Fludrocortisone 0.1 mg bid	112	4.8	140/96
14 weeks	Fludrocortisone 0.1 mg daily	140	5.6	-
5 months	Fludrocortisone 0.1 mg bid	121	4.7	160/100
6 months	Fludrocortisone 0.1 mg daily	152	6.4	-
7 months	Furosemide 20 mg daily	188	5.1	-
8 months	Sodium bicarb. 650 mg tid	160	5.5	-
16 months	Sodium bicarb. 650 mg tid	160	5.2	122/88

Another attempt was made to decrease the fludrocortisone to 0.1 mg daily which was initially tolerable but by 2 months on this dose, her serum potassium was again found to be 6.4 mmol/l with creatinine 152 umol/l. This was transiently treated with higher dose fludrocortisone but the patient felt unwell on the medication, complaining of abdominal pain and so the drug was stopped in favour of furosemide 20 mg daily. Off of the fludrocortisone, her blood pressures were 135/88 but there was resultant hyponatremia, mild hyperkalemia and a sharp rise in serum creatinine to 188 umol/l and so the furosemide was stopped in favour of a return to fludrocortisone once more. Again, her potassium normalized to 4.5 mmo/l with drop in serum creatinine to 127 umol/l. Nephrology was consulted and the patient started on sodium bicarbonate 650 mg tid with cessation of fludrocortisone. During the last 8 months of such therapy, her blood pressures have been normal (122/88), potassium levels have been near-normal (5.2 mmol/l) with stable renal function (creatinine 160 umol/l, eGFR 31 ml/min).

### Case 2

A 60 year old man with a 30 year history of hypertension and serum potassium as low as 2.9 mmol/l was referred for assessment. At presentation he required five antihypertensive agents to control his blood pressure which ranged from 130-150/80-100. His medications were perindopril 8 mg once daily, irbesartan/hydrochlorothiazide 300/25 mg once daily, nifedipine 60 mg twice daily and carvedilol 6.25 mg twice daily. After temporarily stopping carvedilol, irbesartan/hydrochlorothiazide and perindopril, his serum aldosterone was 1041 pmol/l with plasma renin activity of 0.2 ng/l/s yielding an aldosterone to renin ratio (ARR) of 5205 pmol/l/ng/ml/h (normal less than 2000). CT abdomen showed a multinodular left adrenal gland with a 1.3 cm dominant nodule. Adrenal vein sampling (AVS) showed left lateralization with lateralization indices of 4:1 and 12:1 pre and post cosyntropin infusion. Preoperative serum creatinine and GFR were 77 umol/l and 60 ml/min respectively. He underwent left adrenalectomy for 1.5 cm adrenocortical adenoma in the setting of background adrenal hyperplasia. At 4 weeks follow up his blood pressure was 128/80, serum creatinine 77 umol/l, serum aldosterone less than 70 pmol/l with renin activity of 0.31 ng/l/s yielding ARR of less than 225 pmol/l/ng/ml/h. At that time his irbesartan/hydrochlorothiazide was discontinued and nifedipine was reduced to 60 mg daily instead of twice daily and he continued to take carvedilol and perindopril. At 2 months follo up his serum potassium was found to be 6.0 mmol/l and serum creatinine 141 umol/l. He admitted using four pills of Ibuprofen for a fall related injury. At that visit his ACE inhibitor was stopped. A day later his serum creatinine came down to 121 umol/l but potassium remained at 5.9 mmol/l. He was started on fludrocortisone 0.05 mg daily. Two months later his serum creatinine was 115 umol/l and potassium 5.3 mmol/l. He did not have clinical signs of volume depletion and no other apparent reason for high potassium was found; therefore he was switched to furosemide 20 mg once daily. He required loop diuretic to normalize his serum potassium for 6 months after which it was stopped as his serum potassium remained 4.5–4.9 mmol/l without further intervention. At that time his only medications were nifedipine 60 mg daily and carvedilol 6.25 mg daily. At 15 months post-operative, he was successfully weaned off of carvedilol and his only continuing medication was nifedipine 30 mg daily with average blood pressures of 136/90.

### Case 3

A 52 year old woman with type 2 diabetes and congenital right kidney aplasia was referred with a 12 year history of hypertension and hypokalemia as low as 2.3 mmol/l. At presentation she required amlodipine 10 mg once daily, telmisartan 80 mg once daily and spironolactone 25 mg once daily to maintain normotension and eukalmeia. Off of spironolactone, her aldosterone was 1157 pmol/l and direct renin was 3 ng/l yielding ARR of 368 pmol/l/ng/l (normal < 60 using the newer direct renin assay). CT abdomen showed 1.5 cm hypodense left adrenal mass. AVS showed left lateralization with lateralization index of 20:1 pre and post stimulation. Preoperative GFR was 69 ml/min and serum creatinine of 80 umol/l. She underwent adrenalectomy for what was reported as 2 cm adrenal cortical mass with no surrounding hyperplasia. Two weeks after the surgery she presented to emergency department with intermittent nausea and vomiting. Her serum potassium was 6.3 mmol/l and serum creatinine was 150 umol/l. After fluid resuscitation her serum potassium dropped to 5.1 mmol/l but creatinine persisted at 160 umol/l. She was started on sodium bicarbonate 650 mg bid. At 2 weeks follow up her serum creatinine was 208 umol/l and potassium remained at 5.1 mmol/l. She did not require any more antihypertensive medications and her blood pressure was 140/70. She was started on fludrocortisone 0.05 mg once daily and at 1 week follow up lab work her serum creatinine reduced to 170 umol/l with serum potassium of 5.2. She is currently on fludrocortisone 0.05 mg once daily.

## Discussion

Post APA resection hyperkalemia is a well-documented entity, however the incidence and prevalence is not definitively known given the predominant reliance upon case reports and case series for its reporting. As the number of PA diagnoses increase with increasing visibility of the PA literature, it is possible that post APA-resection hyperkalemia will become a more common problem and endocrinologists will need to be familiar with the management options as severe hyperkalemia may be life-threatening.

Three retrospective studies have attempted to define the incidence and risk factors for post-operative hyperkalemia. Chiang et al. studied 55 post-operative APA patients through weekly blood testing and 16 (29.1 %) out of those developed hyperkalemia with serum potassium levels 5.3–6.2 mmol/l within 1 to 3 weeks of surgery [3]. In most cases, the hyperkalemia was transient, resolving by 2 months post-operative but in several cases, the high potassium persisted for longer than 9 months. Results from the German Conn’s Registry [4] showed that of 110 patients, 18 (16 %) developed post-operative hyperkalemia, of whom 12 patients had transient hyperkalemia while in six patients hyperkalemia persisted beyond 11 months. Three out of the six patients required emergency room treatment for serum potassium greater than 6.8 mmol/l. A third study [5] described an incidence of post-operative hyperkalemia approximating 10.5 % (13 out of 124 patients). In that series, 3.2 % had transient hyperkalemia and 7.3 % had hyperkalemia that was prolonged beyond 3 months. Together, these studies from diverse populations demonstrate that hyperkalemia is not rare following APA resection and in a small but meaningful proportion it can be severe and prolonged.

## Risk factors for post-adrenalectomy hyperkalemia

### Older aged patients

Older age seems to correlate with the propensity to develop post-operative hyperkalemia in patients with primary hyperaldosteronism. Subjects aged more than 53 years old had greater odds of developing post-op hyperkalemia than those aged less than 53 years (OR = 15.6) as described in the Korean study [5]. Fischer et al. noted that patients who developed hyperkalemia were significantly older as compared to normokalemic population (65 ± 9 vs. 50 ± 12 years. *P* = 0.026) [4]. This theme is consistent in the study by Chiang et al.: Patients who developed hyperkalemia were older (56.4 ± 4 vs. 46.5 ± 10.0 year. *P* = 0.002). However, in the same study risk factors were analyzed further using multivariate regression and after backward elimination, older age was not one of the independent risk factors [3]. It is possible that older patients had longer duration of hypertension with more advanced hypertensive co-morbidities including hypertensive nephropathy.

### Duration of hypertension

Duration of pre-operative hypertension has been shown to be another potentially important risk factor for developing post adrenalectomy hyperkalemia. In the study by Chiang et al. [3], post-op hyperkalemics had longer duration of hypertension (12.8 ± 9.3 vs. 6.7 ± 5.0 year. *P* = 0.013). Park [5] found that subjects with duration of hypertension greater than 9.5 years had 10 times higher risk of developing hyperkalemia than those with less than 9.5 years of hypertension in an age adjusted model. All of the three subjects presented in our report had a lengthy duration of hypertension (> 10 years) prior to diagnosis and surgery.

### Impaired renal function

Impaired renal function is an important risk factor in predicting post-operative hyperkalemia. The Chiang paper demonstrated that CKD (Stages III to–V) was 30 % more prevalent in patients who ultimately developed hyperkalemia [3]. Park took a more formal approach performing a ROC analysis to determine the most likely cut off values for pre-operative eGFR’s ability to predict post-operative hyperkalemia; an eGFR of < 58.2 ml/min was associated with increased risk of developing hyperkalemia (OR = 26.6 *p* < 0.05) [5]. Fisher found that post-op hyperkalemics had significantly worse pre-operative renal function (creatinine: 91umol/l [76; 114 umol/l] vs. 61umol/l [53; 76 umol/l], *P* = 0.001; GFR: 56 ± 17 vs. 84 ± 21 ml/min, *P* = 0.024) [4].

While overt pre-existing renal impairment may be a strong factor in predicting post-operative hyperkalemia, there is now evidence that PA itself may induce a hyper-filtration injury that may mask renal impairment until the operative reversal of aldosterone mediated hyper-filtration [6]. In the first case presented above, the patient did not have any evidence of renal impairment pre operatively but after APA resection her eGFR decreased until it plateaued at 30–40 ml/min. Thus it may be that a “normal” eGFR (60–100 ml/min) in the PA pre-operative state may actually portend underlying nephropathy that will become evident and clinically relevant in the post-operative period.

Ribstein et al. [7] studied 25 patients with tumoral PA. Renal studies (urinary excretion of proteins, GFR, and effective renal plasma flow [ERPF] were performed both before and 6 months after adrenalectomy. A control group consisting of patients with essential hypertension (EH) was studied before and after 6 months of antihypertensive therapy. At baseline, PA and EH patients were similar with respect to demographic data, duration and level of hypertension, and GFR and ERPF. Urinary excretion of albumin and β2 microglobulin (a marker of glomerular filtration) was higher in PA than EH. Adrenalectomy was followed by a decrease in arterial urinary excretion of albumin and β2 microglobulin, and GFR and ERPF. In EH, a similar decrease in pressure was associated with a decrease in albuminuria but no change in GFR or ERPF. In patients who received a 6 months treatment of spironolactone, both GFR and ERPF decreased in parallel with BP, similar to what was observed after surgery. This data suggests that PA was associated with relative hyperfiltration, unmasked after suppression, removal or blockade of aldosterone excess.

A few studies have attempted to identify predictors of decreasing eGFR after APA resection. Tanase-Nakao et al. [8] studied patients with PA who underwent unilateral adrenalectomy and were followed for 1 month post operatively. Patients who underwent non-PA adrenalectomy were studied as control. Of PA patients, 37.8 % “developed CKD” (defined as eGFR < 60 ml/min/1.73 m2). None of non-PA group developed CKD post op. Of the pre op variables, logistic regression analysis showed that lower pre-surgical eGFR and higher aldosterone-to-renin ratios (ARR) were the independent predictors of post-op CKD in PA. Another Japanese study [6] prospectively described 120 patients with APA who underwent adrenalectomy while 111 patients with bilateral adrenal hyperplasia (BAH) received MR antagonists. Urine albumin excretion (UAE), blood pressure and eGFR decreased significantly at 1 month after treatment in both arms but did not decrease further at 12 months. Multivariate regression analysis revealed that higher UAE and lower serum potassium at initial visit were independent predictors of decreasing eGFR after intervention.

Ribstein et al. [7] showed that baseline serum potassium correlated with the post-operative change in GFR by univariate analysis. Rossi [2] in the PAPY study provided evidence that low serum potassium at baseline predicts albuminuria. Renicke [9] described that hypokalemic PA tended to be associated with renal impairment. Initial potassium concentration (odds ratio, 1.3; 95 % confidence interval, 1.0–5.2; *P* < 0.01), plasma aldosterone concentrations (*P* < 0.05), and presence or absence of the hypokalemic variant of PA (*P* < 0.05) were independent predictors of higher creatinine.

While hypokalemia may simply be a marker of more severe aldosteronism, it must also be considered that hypokalemia could actually play a pathophysiologic role in renal impairment. Low potassium itself can cause functional and structural defects in kidneys, such as cyst formation, tubular vacuolization, interstitial inflammation and gradual loss of renal function [10].

### Post-operative hypoaldosteronism

Connell et al. [11] described a case of a 50 year old woman who developed hyperkalemia and acute kidney injury 1 month after resection of APA. Her aldosterone level was inappropriately low, given hypovolaemia and hyperkalaemia, suggesting hypoaldosteronism (plasma aldosterone 100 pmol/l; plasma renin activity 4.4 pg/ml/h). Gadallah [12] reported a case in which unilateral adrenalectomy for adrenal adenoma was followed by severe hyperkalemia, marked volume depletion and undetectable plasma renin activity and serum aldosterone suggesting chronic suppression of the renin-aldosterone axis. One year later, a gradual return to normokalemia, normotension, and normal plasma renin activity and aldosterone levels was achieved, indicating resolution of the suppression of the renin-aldosterone axis.

In the German Conn’s registry post-operative hyperkalemia caused by suppression of the zona glomerulosa (ZG) insufficiency was examined. They measured serum potassium, post-operative aldosterone and trans-tubular potassium gradient (TTKG) to determine ZG insufficiency which was defined as was defined as undetectable plasma aldosterone level (< 97pmol/l) in the presence of hyperkalemia (K > 5.0). Of the 18 patients who developed hyperkalemia 14 had undetectable and four had low aldosterone levels (aldosterone level < 138 pmol/l). In all six patients who developed persistent hyperkalemia, serum aldosterone level was less than 97pmol/l. The calculation of the TTKG showed decreased values of less than 6, in three of four evaluated hyperkalemic patients, showing inappropriate renal response to hyperkalemia. The normal response to hyperkalemia is an increase in aldosterone secretion leading to an increased urinary potassium excretion, with an increase of TTKG to greater than 10 [13]. A result of less than 6 in a hyperkalemic patient indicates inadequate aldosterone concentration or effect [14]. They also assessed the contralateral gland suppression index in those patients who had selective adrenal vein sampling (AVS) prior to surgery (*n* = 77). Five of five investigated patients with persistent hyperkalemia demonstrated a contralateral aldosterone/cortisol ratio suppression index of aldosterone in the non-dominant gland less than 1 versus inferior vena cava [4].

Taniguchi et al. [15] reported a case of a 46-year-old woman who underwent unilateral adrenalectomy and developed episodes of severe hyperkalemia for 4 months. Plasma aldosterone concentration (PAC) was low (102.5 pmol/l) and undetectable renin activity. Serum potassium levels gradually decreased with concomitant increase in PAC (162pmol/l).

It is plausible that aldosterone synthesis of adjacent and contralateral adrenal glands is severely impaired in some cases with primary hyperaldosteronism, as is glucocorticoid synthesis in Cushing syndrome. Since many instances of hyperkalemia may go undetected post operatively, a larger series with prospective, standardized, sequential potassium measures may be needed in order to better determine if contralateral suppression might predict hyperkalemia.

### Pre-operative mineralocorticoid receptor (MR) antagonists

MR antagonists are often administered in APA patients to control hypokalemia. Reversal of hyperaldosteronism after long term treatment with spironolactone (SP) has been reported in the literature, and it appears that it has anti-steroidogenic effect. Yoneda et al. [16] reported a case of a 41 year old man who had a left sided APA. The plasma renin activity (PRA) was 0.6 ng/ml/h and the plasma aldosterone concentration (PAC) was 362 pg/ml in the supine position. CT showed a 5 mm left sided nodule and AVS indices showed left lateralization. The patient received spironolactone 75 mg OD daily for 5 years and 50 mg for another 4–5 years. At 10 year follow up after discontinuation of spironolactone, the patient’s blood pressure, serum potassium level, and plasma aldosterone concentration remained in the normal range. Although the left adrenal gland tumor was still present on computed tomography after treatment, a furosemide and upright posture test, a captopril challenge test, and a saline loading test produced no evidence of PA. Adrenal vein sampling demonstrated no sign of lateralization.

Demura et al. [17] have shown that the aldosterone synthetic enzyme, CYP11B2, is influenced by epigenetic factors. Long-term treatment with spironolactone may influence the epigenetic modulation of CYP11B2. SP (spironolactone) not only is an MR antagonist, but also forms SP body inclusions in cells after long-term treatment. Histological examination of SP body-containing cells demonstrates enhanced but abortive steroidogenic activity [18]. These observations suggest that MR antagonists, at long exposure times, may actually impair aldosterone production, something that may explain or aggravate the post adrenalectomy hypoaldosterone state.

Park et al. [5] found that out of 124 patients, 93 were treated with spironolactone at a mean dose of 88 mg for mean duration of 45 days. The proportion of MR antagonist users was not significantly different between subjects with persistent hyperkalemia, transient hyperkalemia and normokalemia. Preoperative treatment with MR antagonists did not influence the incidence of hypoaldosteronism and hyperkalemia.

These clinical reports do not yet confirm the role of pre-operative MR antagonists in the development of post-operative hyperkalemia but at the very least should emphasize the importance of immediate post-operative discontinuation of such medications.

## Management of hyperkalemia post adrenalectomy

Post adrenalectomy hyperkalemia can be severe and life threatening, sometimes requiring emergency treatment. Preventative strategies in high risk patients would include restricting dietary potassium intake and a useful patient-oriented website (http://www.kidney.ca/document.doc?id=945. https://www.kidney.org/atoz/content/potassium) may help with patient dietary education. Reversible causes should be addressed in all patients such as hypovolemia, urinary tract obstruction and non-steroidal anti-inflammatory drugs and renin-angiotensin-aldosterone-system inhibitors which can all cause aggravate hyperkalemia in these patients, usually by further decreasing an already impaired GFR. Pharmacological therapies work mainly by potassium removal either by binding it in gut or excreting it through kidneys.

There are several agents which promote renal excretion of potassium. Fludrocortisone is traditionally the obvious choice for hypo-aldosteronism. However, it can be challenging to treat with fludrocortisone in the long term on account of the risk of hypertension and peripheral edema and potentially worsening heart failure in patients with underlying cardiac disease. An alternate approach would use furosemide and oral sodium bicarbonate to increase renal potassium (K) excretion. Studies on the mechanism of distal K secretion have highlighted the importance of renal outer-medullary K (ROMK) and maxi-K channels in distal nephron [19]. Flow dependant K secretion works via apical membrane stretch in the distal nephron, which activates calcium channels, increasing intracellular calcium and activation of maxi-k and ROMK channels to facilitate potassium excretion [20]. Distal Na delivery is however indispensable for K secretion as it provides the electro-chemical gradient driving for K efflux via maxi-K and ROMK. Therefore, an alternative solution to replacing mineralocorticoid is to increase distal nephron flow and sodium delivery via increased Na intake (ideally with NaHCO3), increased water intake and/or loop diuretics or carbonic anhydrase inhibitors. Potassium binders are used to excrete potassium mainly through binding in the gut. A major concern with binders especially sodium polystyrene sulfonate is the development of intestinal necrosis involving colon and ileum [21]. It is a rare event, on the order of 0.2 to 0.3 %. Binders should be avoided in patients with ileus, opiate use and recent abdominal surgery [22]. Therefore these agents are not recommended in the immediate post-adrenalectomy state.

## Clinical approach to management

**Fig. 1:**
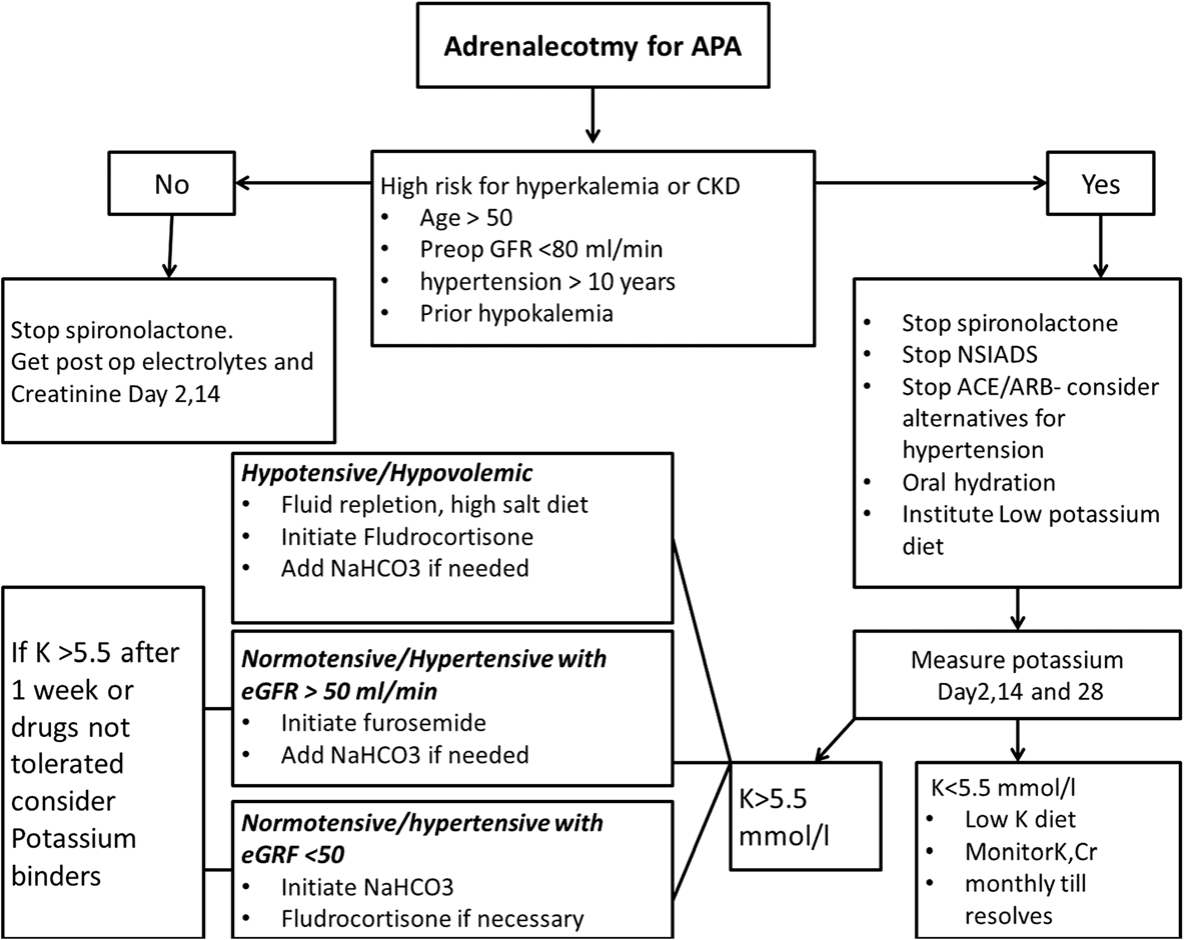
Post-adrenalectomy hyperkalemia- prediction, monitoring and treatment algorithm

Since there are no formal studies or guidelines to inform the best practices in treating hyperkalemia post-adrenalectomy, we propose following prediction, monitoring and treatment strategy outlined in Fig. 1. Vigilance and early intervention with careful follow up testing is the cornerstone of the clinical approach.

## Conclusion

Post APA resection severe hyperkalemia may be a common entity and screening should be actively considered in high risk patients. Older age, longer duration of hypertension, impaired pre-op and post-op GFR and higher levels of pre-op aldosterone and are all risk factors which predict the likelihood of developing post-operative hyperkalemia (see Table 2). Treatment choice should be tailored to patient characteristics including fluid status, blood pressure and serum creatinine (Table 3).

**Table 2:** Known risk factors increasing likelihood of post-operative hyperkalemia

Risk Factors	
1. Older age	
2. Long duration of hypertension	
3. Impaired pre-operative GFR	
4. Impaired post-operative GFR	
5. Higher per-operative aldosterone level

**Table 3:** Pros and cons of different drugs used to treat hyperkalemia

Drugs	Pros	Cons
Fludrocortisone	1. Effective and quick resolution of hyperkalemia 2. Easy titration of dosing 3. Can be used early post operatively	1. Can cause/worsen hypertension 2. Can mask underlying renal disease 3. Can worsen edema, heart failure
Sodium bicarbonate	1. Can be used safely in CKD 2. Can be used early post operatively	1. Unfamiliarity with the drug 2. Not much data available regarding dosing regimen
Loop diuretics	1. Effective and familiar drug 2. Can be used in hypertensive population	1. Can worsen kidney disease by dehydration
Potassium binders	1. Can be used in CKD 2. Does not interfere with blood pressure	1. Can cause complications in post-operative and constipated patients

The long term cure of primary aldosterone excess and hypertension is expected to yield renal benefits but development of irreversible post adrenalectomy renal impairment after a long duration of hypertension may argue for earlier consideration of a PA diagnosis in hypertensive populations. With increasing numbers patients diagnosed with PA and submitted to adrenalectomy, endocrinologists will need to become familiar with the factors that predict severe hyperkalemia and adopt an aggressive approach to prevent life threatening hyperkalemic complications.

## Abbreviations

APA, aldosterone producing adenoma; ARR, aldosterone to renin ratio; BAH, bilateral adrenal hyperplasia; MR, mineralocorticoid receptor; PA, primary aldosteronism; TTKG, trans tubular potassium gradient
